# Beneficial Effect of Lenalidomide-Dexamethason Treatment in Relapsed/Refractory Multiple Myeloma Patients: Results of Real-Life Data From 11 Hungarian Centers

**DOI:** 10.3389/pore.2021.613264

**Published:** 2021-04-22

**Authors:** Gergely Varga, András Dávid Tóth, Virág Réka Szita, Zoltán Csukly, Apor Hardi, Júlia Gaál-Weisinger, Zsolt Nagy, Elvira Altai, Annamária Rencsik, Márk Plander, Tamás Szendrei, Krisztina Kórád, Gáspár Radványi, János Rottek, Beáta Deák, Erika Szaleczky, Tamás Schneider, Zoltán Kohl, Szabolcs Kosztolányi, Hussain Alizadeh, Zsuzsanna Lengyel, Szabolcs Modok, Zita Borbényi, Szilvia Lovas, László Váróczy, Árpád Illés, Péter Rajnics, Tamás Masszi, Gábor Mikala

**Affiliations:** ^1^ Department of Internal Medicine and Haematology, Semmelweis University, Budapest, Hungary; ^2^ National Institute for Hematology and Infectious Diseases, Department of Hematology and Stem Cell Transplantation, South Pest Central Hospital, Budapest, Hungary; ^3^ Department of Internal Medicine and Oncology, Semmelweis University, Budapest, Hungary; ^4^ Veszprém-Csolnoky Ferenc County Hospital, Hematology, Veszprém, Hungary; ^5^ Markusovszky University Teaching Hospital, Szombathely, Hungary; ^6^ Borsod-Abaúj- Zemplén Central County University Teaching Hospital, Miskolc, Hungary; ^7^ National Institute of Oncology, Budapest, Hungary; ^8^ 1st Department of Internal Medicine, University of Pécs, Pécs, Hungary; ^9^ 2nd Department of Medicine and Cardiology Center, University of Szeged, Szeged, Hungary; ^10^ Department of Hematology, Institute for Medicine, Clinical Center, University of Debrecen, Debrecen, Hungary; ^11^ Somogy County Kaposi Mór Teaching Hospital, Kaposvár, Hungary

**Keywords:** myeloma, lenalidomide, relapsed, real life, treatment

## Abstract

In Hungary, the cost of lenalidomide-based therapy is covered only for relapsed multiple myeloma (MM) patients, therefore lenalidomide is typically used in the second-line either as part of a triplet with proteasome inhibitors or as a doublet. Lenalidomide-dexamethasone is a standard treatment approach for relapsed/refractory MM, and according to recent large randomized clinical trials (RCT, the standard arm of POLLUX, ASPIRE, TOURMALINE), the progression-free survival (PFS) is expected to be approximately 18 months. We surveyed ten Hungarian centers treating MM and collected data of 278 patients treated predominantly after 2016. The median age was 65 years, and patients were distributed roughly equally over the 3 international staging system groups, but patients with high risk cytogenetics were underrepresented. 15.8% of the patients reached complete response, 21.6% very good partial response, 40.6% partial response, 10.8% stable disease, and 2.5% progressed on treatment. The median PFS was unexpectedly long, 24 months, however only 9 months in those with high risk cytogenetics. We found interesting differences between centers regarding corticosteroid type (prednisolone, methylprednisolone or dexamethasone) and dosing, and also regarding the choice of anticoagulation, but the outcome of the various centers were not different. Although the higher equivalent steroid dose resulted in more complete responses, the median PFS of those having lower corticosteroid dose and methylprednisolone were not inferior compared to the ones with higher dose dexamethasone. On multivariate analysis high risk cytogenetics and the number of prior lines remained significant independent prognostic factors regarding PFS (*p* < 0.001 and *p* = 0.005). Our results show that in well-selected patients Lenalidomide-dexamethasone can be a very effective treatment with real-world results that may even outperform those reported in the recent RCTs. This real world information may be more valuable than outdated RCT data when treatment options are discussed with patients.

## Introduction

Lenalidomide in combination with dexamethasone (Rd) has been widely used in relapsed/refractory multiple myeloma (R/R MM) for more than a decade and is now the backbone of most three and four-drug combinations for both newly diagnosed and relapsed myeloma. Nevertheless, it is difficult to tell exactly what to expect currently when lenalidomide is used as a doublet in relapsed patients. Based on the results of two pivotal randomized clinical trials (MM-009 and MM-010), the Food and Drug Administration approved Rd for the treatment of MM patients who have received at least one prior line of therapy in 2006 [1–3], however, the landscape has changed immensely since then, with lenalidomide being increasingly used in the first-line setting. As the treated cohort changed, so did the outcome, as demonstrated nicely by some recent randomized clinical trials (RCTs, e.g. POLLUX, ASPIRE, TOURMALINE), where Rd was used in the control arm with progression-free survivals (PFS) significantly longer than in the aforementioned trials [4–6].

As lenalidomide-based therapy is only funded for relapsed patients in Hungary, most patients receiving second-line therapy are lenalidomide-naïve, and thus treated with either Rd or an Rd based triplet. There are however still many open questions regarding its optimal use: whether we can use Rd rather than a triplet without significantly compromising our patients’ outcome, and what results we may expect from this protocol.

In this study, we approached all Hungarian hematology facilities treating MM patients with a questionnaire about their use of Rd in the routine clinical setting, collecting data with regards to patient characteristics and associated treatment outcome and compared it with results from the initial phase 3 clinical trials as well as with the control arm of some recent RCTs of Rd backbone triplets.

## Patients and Methods

### Patients

Eleven Hungarian centers responded to our query, supplying data about 283 patients treated with Rd over the course of the last 10 years from 2010–19, however only eight patients were started on Rd before 2015, eight in 2015, all ther others after 2015, as the need for individual funding applications had held back its widespread use until then. Post autologous stem cell transplantation (ASCT) lenalidomide maintenance was not used in this group of patients as it was not funded until 2018. Patients treated with Rd in the first line, or with Rd used in a triplet were excluded, we collected data about later “add-on” use of third drugs. Prognostic markers such as ISS and fluorescence *in situ* hybridization (FISH) have been collected as well as the response according to the International Myeloma Working Group (IMWG) criteria and progression-free and overall survivals (OS) [7, 8]. High-risk FISH result was defined as t (4; 14), t (14; 16) and/or del (17p), per the published IMWG guideline [9], the threshold for del (17p) was 5%.

### Statistical Analysis

The primary endpoint was PFS and secondary outcome measures were overall response rate and OS. PFS was measured from the start of treatment to the date of disease progression or death. The overall response rate was defined as the collective proportion of pts with complete response (CR), very good partial remission (VGPR), partial remission (PR), or minimal response (MR) as their best response. OS was defined as the time from the start of treatment to the date of death. Comparisons of dichotomous variables were performed by Fisher’s exact test. Continuous variables were compared by Mann–Whitney or Kruskal–Wallis tests. PFS and OS were estimated by Kaplan–Meier analysis, baseline clinical characteristics were evaluated for predictive significance by multivariate Cox regression. The analyses were carried out using the SPSS (version 20.0; SPSS, Chicago, IL) software package.

## Results

### Patient Characteristics

We analyzed the data of 283 patients treated with Rd at 11 centers. An overview of patient characteristics is presented in Table 1. The median age of the patients at diagnosis (65 years) was typical for the Hungarian myeloma cohort, however, the age at Rd initiation (70 years) was higher than that of the typical relapsed patient in clinical trials. The number of patients with known high-risk features (high ISS, HR FISH, extramedullary disease, and renal failure) was lower in each category than expected. HR FISH included t (4; 14), t (14; 16) and del (17p); 24 additional patients had amplification of chromosome 1q21. The performed prognostic tests varied widely between centers, with some doing FISH routinely while others not, also taking into account that plasma cell selection had not universally been routine before 2018.

**TABLE 1:** Patients’ characteristics.

Total number/male/female	283/140/143
Median and mean age at diagnosis in years (range)	65.2/64.1 (28.2–86.3)
Median and mean age at Rd initiation in years (range)	70.2/68.4 (36.1–90)
Heavy chain IgG, A, LC, non-secretory (%)	62.9/22.4/12.1/1.4
Light chain kappa, lambda (%)	66.9/31.6
ISS at diagnosis (ISS 1/2/3%; missing 45)	30.4/37.1/32.5
FISH (SR/HR %; missing 100)	87.5/12.5
ISS pre Rd (ISS 1/2/3%; missing 110)	36.6/40.0/23.4
Extramedullary disease pre Rd (%)	9.1
Renal failure (GFR <30 ml/min, %)	12.8
Median prior lines (range)	2 (1–8)
Prior thal/bor/len (%)	65.6/92.4/3.2
Prior ASCT (%)	46.5
Steroid: dex, methylpred (%)	62.7, 37.3
Median weekly dex eqvivalent^a^ corticosteroid dose (<20 mg/20 mg/40 mg %)	14.7/36.8/48.5

^a^ 1 mg dexamethasone is roughly equivalent to 5 mg methylprednisolone.

Abbreviations: ASCT, autologous stem cell transplantation; bor, bortezomib; dex, dexamethasone; FISH, fluorescence *in situ* hybridization; ISS, international staging system; len, lenalidomide; methylpred, methylprednisolone; Rd, lenalidomide-dexamethasone; thal, thalidomide.

### Response

Out of the 259 patients who had response assessment, 20.5% had CR, 25.9% VGPR, 36.7% PR, 14.5% SD and only 2.4% PD. ISS, HR cytogenetics did not affect the best response (data not shown). Similarly, patients with renal failure had a comparable response rate to those without, while patients with extramedullary disease had a trend to have less than PR (26.1 vs. 13.1%, *p* = 0.09). Unsurprisingly, the chance of reaching a good response declined with more than 3 prior lines (data not shown), however, previous exposure to thalidomide, bortezomib, lenalidomide, or ASCT did not affect the response.

### Addition of 3rd Drug

An interesting sub-analysis looked into the 54 patients where the treating physician escalated the therapy with the addition of a third drug after a median of two months due to suboptimal response (PD 7.8%, SD 41.2%, PR 41.2%). Keeping in mind that with Rd as a doublet you can expect a deepening response at this stage too, some of these patients experienced an upgrade of their initial response after the third drug was added (PR patients: 35% CR, 30% VGPR, 35% PR; SD patients 14.3% CR, 19% VGPR, 28.6% PR, 38.1% SD; PD patients 25% VGPR, 75% PD). The third drug was bortezomib in 21, cyclophosphamide in 3, bendamustine in 3, carfilzomib in 2, daratumumab in 5, and ixazomib in 20 cases (this latter probably due to its availability in a compassionate use program).

### Survival

**FIGURE 1:**
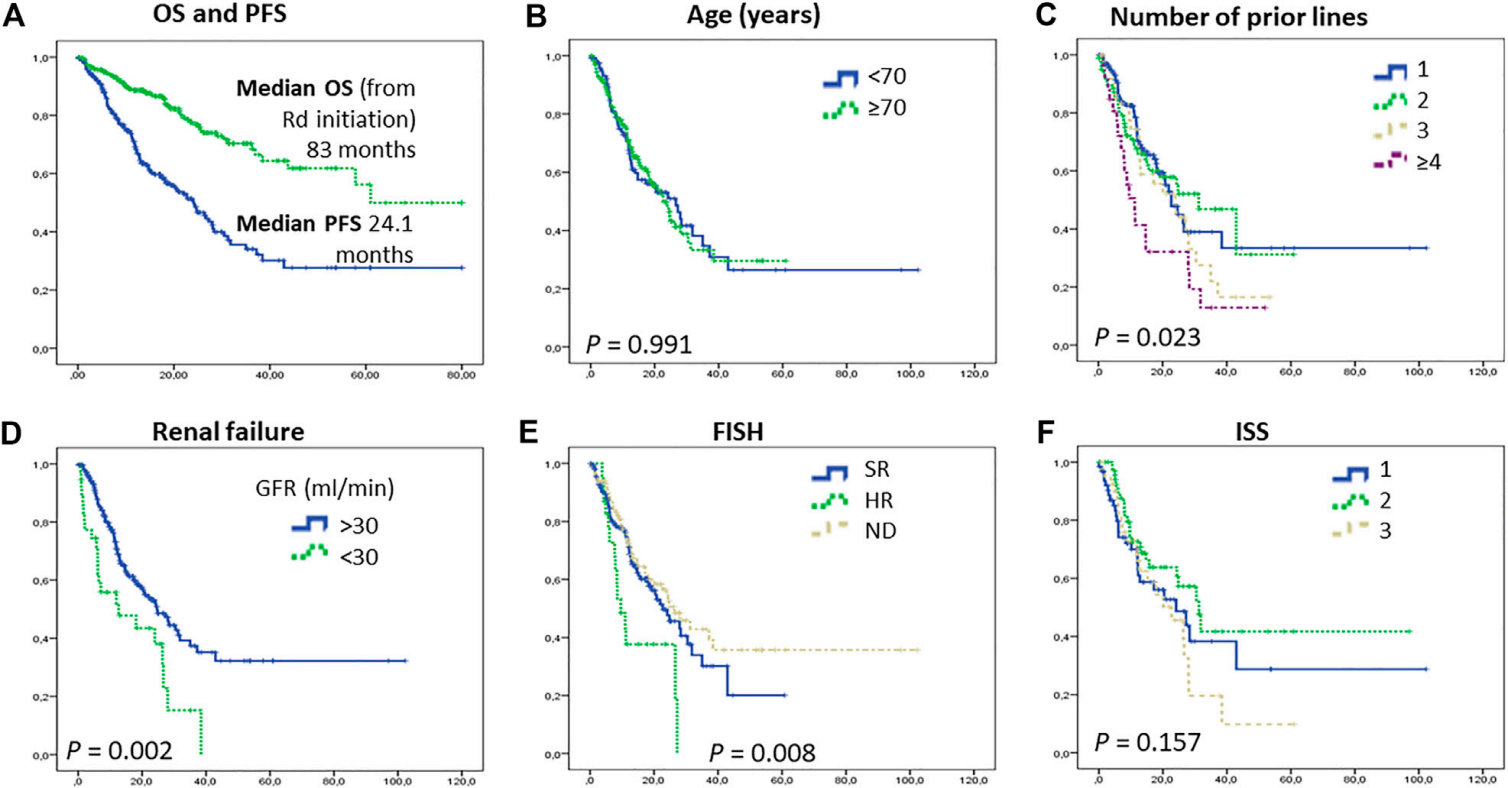
Survival of patients. Overall and progression-free survival of the whole cohort **(A)**; Progression-free survival according to Age **(B)**, number of prior lines **(C)**, renal function **(D)**, FISH **(E)**, and ISS **(F)**. Both PFS and OS have exceeded what was expacted based on the pivotal randomized clinical trials [2]. Patients with renal failure and high risk cytogenetics were benefited less from Rd treatment. Abrreviations: FISH, fluorescence *in situ* hybridization; GFR, glomerular filtration rate; ISS, international staging system; OS, overall survival; PFS, progression free survival; Rd, lenalidomide-dexamethason.

The median progression-free survival (PFS) of the whole group was 24.3 months, the overall survival (OS) calculated from the start of the Rd protocol 83.0 months (Figure 1A). The PFS was similar in patients above and below 70 years of age (22.7 and 26.3 months, *p* = 0.36, Figure 1B), the survival of patients with 1, 2 and 3 prior therapies was similar, however above 3 lines the PFS was significantly worse (1 line 22.7, 2 lines 31.7, 3 lines 24.0 months, 4 or more lines 11.3 months, *p* = 0.023, Figure 1C). There was no difference between the PFS and OS patients treated in different centers (data not shown).

**FIGURE 2:**
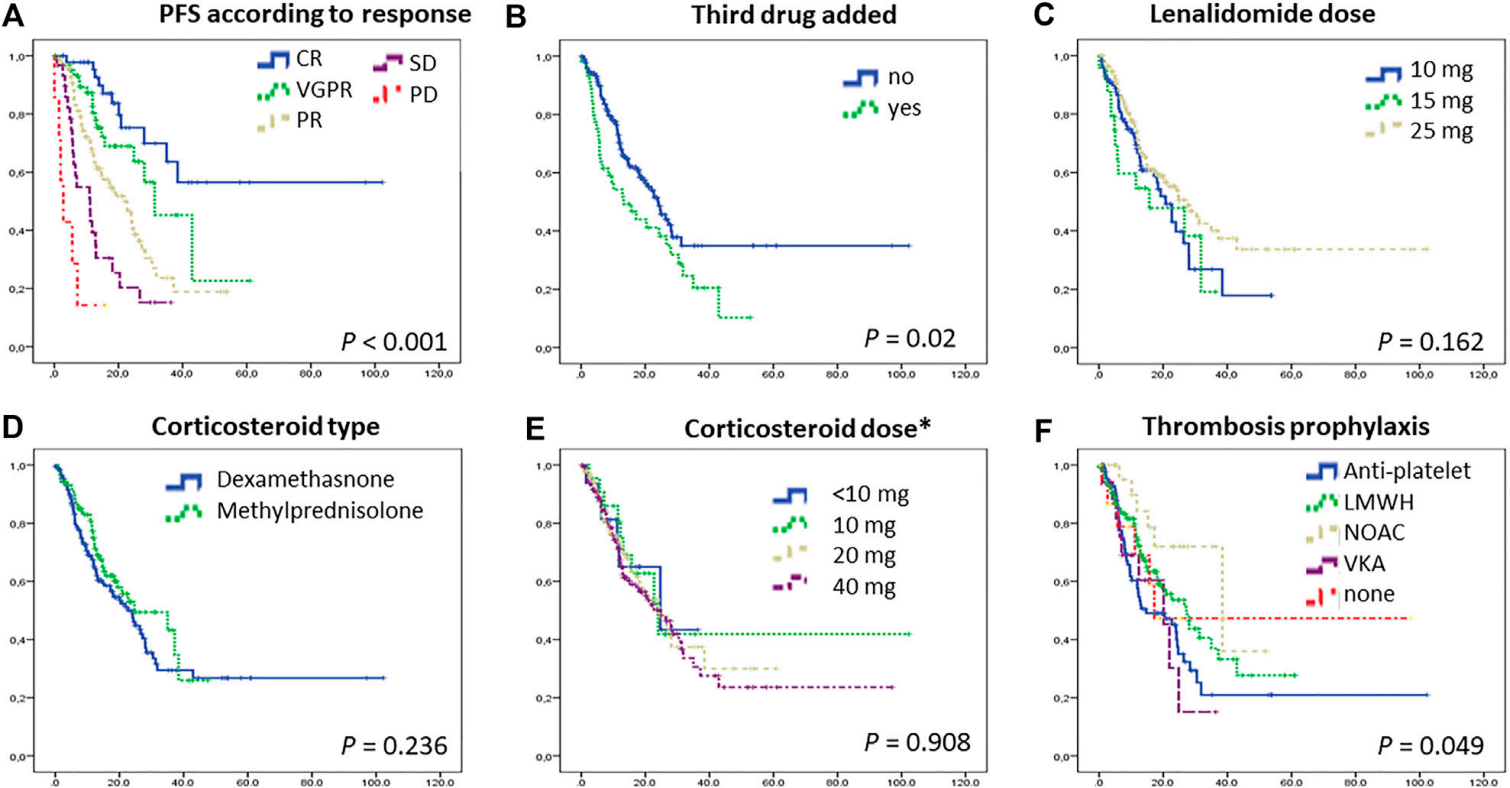
Progression-free survival according to response **(A)**, whether a third drug was added **(B)**, lenalidomide dose **(C)**, cordicosteroid type **(D)** and dose **(E)**, and thromboprophylaxis type **(F)**. Patients with deeper responses had longer PFS. In some cases with suboptimal response, a third drug (usually bortezomib) was added to Rd, but the outcome of these patients remained inferior compared to others. Importantly, the corticosteroid type (dexamethasone or methylprednisolone-a frequently used replacement of dexamethasone in Hungary) and dose did not have an effect on PFS. Abrreviations: CR, complete response; LMWH, low-molecular-weight heparin; NOAC, novel oral anticoagulants; PD, progressive disease; PFS, progression free survival; PR, partial response; Rd, lenalidomide-dexamethason; SD, stable disease; VGPR, very good partial response; VKA, vitamin K antagonists. *dexamethasone (5 mg methylprednisolone is equivalent with 1 mg dexamethasone).

Patients with renal failure prior to Rd initiation had a worse outcome (PFS 12.7 vs. 24.6 months, *p* = 0.002), Those with high-risk FISH also had a significantly worse PFS (8.4 vs. 22.7 months, *p* = 0.008). Interestingly a significant sized cohort of patients with unknown FISH had a similar outcome to those with standard-risk (26.3 months); most likely showing the treating physicians’ more active approach to biopsy patients progressing aggressively and a more laid-back attitude to those behaving suggesting standard risk. ISS assessed at treatment initiation did not predict the PFS duration that was 24.1, 31.3, and 22.7 months in the ISS 1, 2, and 3 groups, respectively (Figures 1D–F). Characteristically, those with a good response to treatment had a significantly better PFS (Figure 2A).

When all the pre-Rd prognostic variables (age, ISS,FISH, renal failure, the number of prior lines) and treatment characteristics (lenalidomide dose, steroid type and dose) were entered to Cox multivariate analysis, only FISH and the number of prior lines remained significant independent factors (*p* < 0.001 and *p* = 0.005).

Those patients whose original response was considered suboptimal, and therefore a third drug was added, fared worse compared to others (Figure 2B). However if those with ixazomib addition (as part of a compassionate use program available at the period) were examined separately, that group had actually a superior PFS.

If the group that has an unexpectedly long (>18 months) survival is examined separately, that cohort has fewer patients with IgA M-protein, less with light chain only MM, marginally more ISS 1 and less ISS 3 patients (*p* = 0.065). From the FISH point of view, 30.6% of the standard risk patients were in this group, while only 17.4% of the high risk. Similarly, from those with 1q amplification, only 15.4% were in this group, while 35.2% of those without were. Renal failure, prior therapy, steroid type, and dose did not play a role here, and interestingly, the response reached at either 2- or 6-months time points did not affect this.

### Lenalidomide Dosing

Lenalidomide dose was adjusted for renal function and age, and physicians used several dosing patterns including 25 mg every other day, 15 mg, and 10 mg daily, however, 55% of the patients in this study had 25 mg lenalidomide, daily (21/28 days, though the treatment holiday period was frequently longer than the standard 7 days, data not shown). The PFS did not differ significantly between these dosing subgroups (Figure 2C).

### Steroid Type and Dosing

An interesting part of our real-world analysis was the comparison of the two types of corticosteroids used by Hungarian centers. In Hungary, dexamethasone is often replaced with methylprednisolone in daily routine as oral dexamethasone has not been marketed and therefore had to be purchased via hospital pharmacies, whereas methylprednisolone is an oral prescription drug available in several different formats. Even still, almost two-thirds of the patients were treated with oral dexamethasone, a minority with intravenous dexamethasone, and slightly more than one third with oral methylprednisolone. The weekly dose was 40 mg dexamethasone in the majority, but lower than that in a significant group. Calculated to the respective equivalent dexamethasone dose, some patients had as low as 4 mg weekly (5.9%), others 10 mg (8.1%), however, the majority had either 20 mg (36.8%) or 40 mg (48.5%). Dose adjustments during therapy were not collected. The preference of corticosteroid type and dose varied according to the treating center. Interestingly, neither the type nor the dose seemed to significantly affect the outcome (Figures 2D,E).

### Type of Thrombosis Prophylaxis

The nature of our data collection did not allow us to readily identify the reasoning behind the choice of thromboprophylaxis in a given patient, but we could notice different trends between the treating centers: some seemed to use antiplatelet therapy in younger patients and anticoagulation (low molecular weight heparin, novel anticoagulants or vitamin K antagonists) in older ones, whereas other centers had a rather uniform preference toward one or another type. In terms of PFS, those on novel anticoagulants had a marginally longer PFS, but this was probably more of an association with better renal function rather than a true causality (*p* = 0.049, Figure 2F).

### Adverse Events

Table 2 shows the adverse events (AEs), as reported. Grading is not included as it was not uniformly provided by the treating centers. The number of AEs here is lower compared to prospective clinical trials, not unusual in retrospective patient chart-type data collections. Only 6 cases of deep vein thromboses were reported, 4 in low-molecular-weight heparin treated patients, and 2 in those taking aspirin. The other common AEs were bone marrow suppression, infections, and diarrhea, none of them requiring permanent treatment discontinuation.

**TABLE 2:** Adverse events. As reported by the treating physcians, number of occurances and proportion of occurances, no gradings were collected. 283 patitent were treated in total, 69 has one or more adverse events reported.

Adverse event	Number of cases and relative proportion (%)
Bone marrow suppression	29 (10.3)
Gastrointestinal	10 (3.5)
Infection	8 (2.8)
Neuropathy	7 (2.5)
Deep vein thrombosis	6 (2.1)
Skin reaction	5 (1.8)
Cardiovascular	2 (0.7)
Fatigue	2 (0.7)
Orthostatic hypotension	2 (0.7)
Vasculitis	1 (0.3)
Acute myocardial infarction	1 (0.3)
Intolerance	1 (0.3)
Periorbital edema	1 (0.3)
Suicide	1 (0.3)

## Discussion

While real-life results of oncology drugs are typically inferior when compared to RCTs, in this case, we found the opposite. According to the later published pooled analysis of the long term survival of the two similarly designed pivotal Rd trials (MM-009 and MM-010), the PFS was only 11.1 months, which of course compared very favorably to the placebo + dexamethason control arm (4.6 months) [1–3]. The patients of these pivotal trials were mostly treated by 2 or more lines of treatment, but only a minority had prior bortezomib (7.6%), and less than half had thalidomide exposure (36%). One could expect that in a novel-agent-naïve population a new drug could work even better, although the opposite is possible as well if the predominantly chemo-treated population was already damaged by ineffective chemotherapy courses giving rise to more resistant clones.

The level of supportive care has also increased significantly since then, probably further contributing to the improved results. Firstly, thromboembolic events were significantly more common in patients treated with lenalidomide plus dexamethasone in the absence of prophylactic anticoagulation which was not recommended at the time. In a later study, IOM-0810 a greater percentage of patients was anticoagulated, but this was still not a mandatory measure [10].

Another important difference is the corticosteroid use. At the time when the pivotal trials were designed, 40 mg oral dexamethasone on days 1–4, 9–12, and 17–20 of each 28-days cycle was recommended. This rather high dose was later proven to be more toxic than the now-standard lower doses [11]. These data resulted in the new and universally approved standard dose of weekly 40 mg (20 mg in more elderly patients). Even more contemporary results advocate further dose reduction of lenalidomide and complete abolition of dex after 9 cycles of upfront Rd treatment [12]. As our results demonstrated, in day to day practice the treating physicians do utilize an even wider range of corticosteroid types and doses depending on patients’ performance status. Importantly, this did not seem to adversely impact the outcome, raising the question of whether it is justified to use 40 mg uniformly in everyone as some centers do, or whether dose adjustment is more appropriate.

The other datasets we can compare our findings to are the standard arm of the recent RCTs using Rd backbones to test the addition of daratumumab (POLLUX, [4], carfilzomib (ASPIRE, [5], or ixazomib (TOURMALINE, [6], which reported PFS as 17.5, 17.6 and 14.7 months respectively, which are again shorter than what our real-world analysis showed. The explanation ought to lie in the obvious difference in patient characteristics: in the RCTs, patients were randomized to have Rd instead of the triplet, whereas off-trial the treating physician could freely select patients with disease of lower perceived risk to receive the doublet treatment. It was outside of the scope of this analysis to review what other treatments were utilized at the time by the centers, but based on the low patient numbers with high-risk cytogenetics in our cohort (this was around 25% in the three RCTs quoted above, and not stated in the pivotal MM-009 and MM-010 trials) we can speculate that high-risk patients had either bortezomib based protocols or other triplets, instead of the Rd doublet.

A cohort of patients with a suboptimal initial result had a 3rd drug added to enhance the effect of the Rd protocol after a median of 2 months. It is difficult to assess in retrospect what exactly the trigger for the upgrade was, but certainly, these patients fared worse compared to the rest, proving that when Rd seems to fail, Rd backbone triplets may not be the best salvage options.

In summary, our results confirmed that Rd remains a very effective treatment in well-selected patients, mostly those who are lenalidomide-naïve and do not show high-risk features. During the COVID-19 pandemic choosing an all-oral combination can be a good alternative to the more expensive, in part intravenous triplets which do require frequent hospital visits exposing the patients to potential encounters with others, spreading the infection.

## Data Availability

The original contributions presented in the study are included in the article/Supplementary Material, further inquiries can be directed to the corresponding author.

## References

[1] Dimopoulos M Spencer A Attal M Prince HM Harousseau J-L Dmoszynska A Lenalidomide Plus Dexamethasone for Relapsed or Refractory Multiple Myeloma. N Engl J Med (2007) 357:2123–32. 10.1056/nejmoa070594 18032762

[2] Dimopoulos MA Chen C Spencer A Niesvizky R Attal M Stadtmauer EA Long-term Follow-Up on Overall Survival from the MM-009 and MM-010 Phase III Trials of Lenalidomide Plus Dexamethasone in Patients with Relapsed or Refractory Multiple Myeloma. Leukemia (2009) 23:2147–52. 10.1038/leu.2009.147 19626046

[3] Weber DM Chen C Niesvizky R Wang M Belch A Stadtmauer EA Lenalidomide Plus Dexamethasone for Relapsed Multiple Myeloma in North America. N Engl J Med (2007) 357:2133–42. 10.1056/nejmoa070596 18032763

[4] Dimopoulos MA Oriol A Nahi H San-Miguel J Bahlis NJ Usmani SZ Daratumumab, Lenalidomide, and Dexamethasone for Multiple Myeloma. N Engl J Med (2016) 375:1319–31. 10.1056/nejmoa1607751 27705267

[5] Stewart AK Rajkumar SV Dimopoulos MA Masszi T Špička I Oriol A Carfilzomib, Lenalidomide, and Dexamethasone for Relapsed Multiple Myeloma. N Engl J Med (2015) 372:142–52. 10.1056/nejmoa1411321 25482145

[6] Moreau P Masszi T Grzasko N Bahlis NJ Hansson M Pour L Oral Ixazomib, Lenalidomide, and Dexamethasone for Multiple Myeloma. N Engl J Med (2016) 374:1621–34. 10.1056/nejmoa1516282 27119237

[7] Durie BGM Harousseau JL Harousseau J-L Miguel JS Bladé J Barlogie B International Uniform Response Criteria for Multiple Myeloma. Leukemia (2006) 20:1467–73. 10.1038/sj.leu.2404284 16855634

[8] Kyle RA Rajkumar SV Criteria for Diagnosis, Staging, Risk Stratification and Response Assessment of Multiple Myeloma. Leukemia (2008) 23:3–9. 10.1038/leu.2008.291 18971951 PMC2627786

[9] Palumbo A Avet-Loiseau H Oliva S Lokhorst HM Goldschmidt H Rosinol L Revised International Staging System for Multiple Myeloma: A Report from International Myeloma Working Group. Jco (2015) 33:2863–9. 10.1200/jco.2015.61.2267 PMC4846284 26240224

[10] Knauf W Aldaoud A Losem C Mittermueller J Neise M Niemeier B Lenalidomide Plus Dexamethasone for Patients with Relapsed or Refractory Multiple Myeloma: Final Results of a Non-interventional Study and Comparison with the Pivotal Phase 3 Clinical Trials. Leuk Res (2018) 68:90–7. 10.1016/j.leukres.2018.03.008 29579627

[11] Rajkumar SV Jacobus S Callander NS Fonseca R Vesole DH Williams ME Lenalidomide Plus High-Dose Dexamethasone versus Lenalidomide Plus Low-Dose Dexamethasone as Initial Therapy for Newly Diagnosed Multiple Myeloma: an Open-Label Randomised Controlled Trial. Lancet Oncol (2010) 11:29–37. 10.1016/s1470-2045(09)70284-0 19853510 PMC3042271

[12] Larocca A Salvini M Gaidano G Cascavilla N Baldini L Glietta M Sparing Steroids in Elderly Intermediate Fit Newly Diagnosed Multiple Myeloma Patients Treated with a Dose/schedule Adjusted RD-R vs. Continuous RD: Results of RV-MM-PI-0752 Phase III Randomized Study: PF586. Hema Sphere (2019) 3(S1):244. Abstract retrieved from EHA Library. 06/14/19; 266385. 10.1097/01.HS9.0000560632.24271.d7

